# The Role of Death Anxiety in Older Adult PTSD, Depression, and Anxiety During Wartime

**DOI:** 10.1093/geroni/igaf122.647

**Published:** 2025-12-31

**Authors:** Yoav Bergman, Rotem Saar-Ashkenazy, Yifat Faran, Eyal Klonover, Yuval Palgi

**Affiliations:** Ashkelon Academic College, Ashkelon, HaDarom, Israel; Ashkelon Academic College, Ashkelon, HaDarom, Israel; Ashkelon Academic College, Ashkelon, HaDarom, Israel; Ashkelon Academic College, Ashkelon, HaDarom, Israel; University of Haifa, Haifa, Hefa, Israel

## Abstract

The outbreak of the Israel–Hamas war on October 7, 2023, has presented unprecedented challenges to older adults’ mental health, including increased posttraumatic stress, anxiety, and depression. The current study examined potential war- and age-related factors associated with probable posttraumatic stress disorder (PTSD), depression, and anxiety among Israeli older adults during the ongoing war. Moreover, due to the continued threat of death, we examined whether death anxiety is an additional contributing factor to older adults’ probable PTSD, depression, and anxiety. Data were collected January–March 2024 from 554 community-dwelling older adults (Mage= 73.90 years, SD = 7.35, range: 61–96 years) who completed online scales assessing sociodemographic variables, war exposure (distance from the Gaza Strip, exposure to terror attacks/blasts), and age-related constructs (assistance in daily activities [ADL], cognitive decline, physical illnesses, death anxiety). Increased ADL was associated with probable depression (B = 0.62, OR = 1.87), and anxiety (B = 0.42, OR = 1.53), and cognitive decline was associated with probable depression (B = 1.52, OR = 4.56). Older adults with high levels of death anxiety were almost 3 times as likely to meet the criteria for probable PTSD (B = 1.05, OR = 2.85) and more than 1.5 as likely to meet the criteria for probable depression (B = 0.54, OR = 1.71) and anxiety (B = 0.50, OR = 1.65). The importance of death anxiety as a potential risk factor for negative psychological outcomes among older adults during war will be discussed.

